# *In Silico* Investigation of Potential PARP-1 Inhibitors from Traditional Chinese Medicine

**DOI:** 10.1155/2014/917605

**Published:** 2014-04-30

**Authors:** Kuan-Chung Chen, Mao-Feng Sun, Calvin Yu-Chian Chen

**Affiliations:** ^1^School of Pharmacy, China Medical University, Taichung 40402, Taiwan; ^2^School of Chinese Medicine-Acupuncture Science, China Medical University, Taichung 40402, Taiwan; ^3^Department of Biomedical Informatics, Asia University, Taichung 41354, Taiwan; ^4^School of Medicine, College of Medicine, China Medical University, Taichung 40402, Taiwan

## Abstract

Poly(ADP-ribose) polymerases (PARPs) are nuclear enzymes which catalyze the poly-ADP-ribosylation involved in gene transcription, DNA damage repair, and cell-death signaling. As PARP-1 protein contains a DNA-binding domain, which can bind to DNA strand breaks and repair the damaged DNA over a low basal level, the inhibitors of poly(ADP-ribose) polymerase 1 (PARP-1) have been indicated as the agents treated for cancer. This study employed the compounds from TCM Database@Taiwan to identify the potential PARP-1 inhibitors from the vast repertoire of TCM compounds. The binding affinities of the potential TCM compounds were also predicted utilized several distinct scoring functions. Molecular dynamics simulations were performed to optimize the result of docking simulation and analyze the stability of interactions between protein and ligand. The top TCM candidates, isopraeroside IV, picrasidine M, and aurantiamide acetate, had higher potent binding affinities than control, A927929. They have stable H-bonds with residues Gly202 and, Ser243 as A927929 and stable H-bonds with residues Asp105, Tyr228, and His248 in the other side of the binding domain, which may strengthen and stabilize ligand inside the binding domain of PARP-1 protein. Hence, we propose isopraeroside IV and aurantiamide acetate as potential lead compounds for further study in drug development process with the PARP-1 protein.

## 1. Introduction

Poly(ADP-ribose) polymerases (PARPs) are nuclear enzymes which catalyze the poly-ADP-ribosylation to combine one or more ADP-ribose moieties from intracellular nicotinamide adenine dinucleotide (NAD^+^) covalently with target proteins [1–3]. The poly-ADP-ribosylation is commonly involved in gene transcription, DNA damage repair, and cell-death signaling [4–6].

There are six domains in the structure of poly(ADP-ribose) polymerase 1 (PARP-1) protein elucidated by recent structural studies. Two of three zinc-binding domains have the function to detect and bind to DNA breaks and the third zinc-binding domain coordinates DNA-dependent enzyme activation [7]. The automodification domain serves as acceptors of ADP-ribose moieties, which allow PARP-1 protein mediated poly-ADP-ribosylation to itself, and contains a BRCA1 C-terminus repeat motif [8–10]. The C-terminal catalytic domain catalyzes the poly-ADP-ribosylation to combine one or more ADP-ribose moieties from intracellular nicotinamide adenine dinucleotide (NAD^+^) covalently with target proteins [11–13]. As PARP-1 protein contains a DNA-binding domain, which can bind to DNA strand breaks and repair the damaged DNA over a low basal level, the inhibitors of poly(ADR-ribose) polymerase 1 (PARP-1) have been indicated as the agents treated for cancer [14–17].

Nowadays, the researchers devote to determining the mechanism of diseases and detecting the useful target protein against the diseases [18–24]. In previous researches, it was proven that many compounds extracted from traditional Chinese medicine (TCM) can be recognized as potential lead compounds treated for viral infection [25–28], stroke prevention [29–31], cancers [32–35], and metabolic syndrome [36–38]. To improve drug development from TCM compounds, this study employed the compounds from TCM Database@Taiwan for virtual screening to identify the potential PARP-1 inhibitors from the vast repertoire of TCM compounds. As the structural disorders of protein may cause the side-effect or affect the ligand binding [39, 40], the prediction of disordered amino acids of PARP-1 protein was performed before docking simulation. In docking simulation, distinct scoring functions had been created to predict the binding affinities in different measure methods, such as LigScore considering the Van der Waals interaction and buried polar surface area, piecewise linear potential (PLP), and potential of mean force (PMF) measuring the pairwise interactions of hydrogen bond (H-bond) and steric interaction. We identify the potential TCM compounds in docking simulation utilizing those scoring functions and dock score, which evaluated the docking poses by interaction energy. Moreover, the molecular dynamics (MD) simulations were performed to optimize the result of docking simulation and analyze the stability of interactions between protein and ligand under dynamic conditions.

## 2. Materials and Methods

### 2.1. Data Collection

The X-ray crystallography structure of human poly(ADP-ribose) polymerase 1 (PARP-1) with A927929 was obtained from RCSB protein data bank with PDB ID: 3L3 M [41]. The crystal structure of PPAR protein was prepared by prepare protein module in Discovery Studio 2.5 (DS2.5) to remove crystal water, protonate the structure of protein, and employ chemistry at HARvard macromolecular mechanics (CHARMM) force field [42]. The binding site of PARP-1 protein was defined by the volume and location of the cocrystallized compound, A927929. A total of 9,029 nonduplicate TCM compounds from TCM Database@Taiwan [43] were filtered by Lipinski's rule of five [44] and protonate the structure by prepare ligand module in DS2.5. The prediction of disordered amino acids of PARP-1 protein was performed by PONDR-Fit [45].

### 2.2. Docking Simulation

The TCM compounds were virtually screened by LigandFit protocol [46] in DS 2.5 to dock compounds into binding site using Monte-Carlo ligand conformation generation and a shape-based initial docking. The suitable docking poses were then optionally minimized with CHARMM force field [42], and a set of scoring functions were evaluated by LigandFit protocol [46] in DS 2.5.

### 2.3. Molecular Dynamics Simulation

The molecular dynamics (MD) simulations are performed by Gromacs [47]. The PARP-1 protein was reprepared with charmm27 force field by Gromacs. The topology and parameters of each ligand for use with Gromacs were provided by SwissParam program [48]. The whole system involves a cubic box with a minimum distance of 1.2 Å from the protein-ligand complex was solvated by a water model of TIP3P. At the beginning of MD simulation, an energy minimization was performed using steepest descent algorithm [49] with a maximum of 5,000 steps and followed by a single 10 ps constant temperature (NVT ensemble) equilibration performed using Berendsen weak thermal coupling method. The total of 40 ns production simulation was performed under the particle mesh Ewald (PME) option with a time step of 2 fs. The 40 ns MD trajectories were analyzed by the protocols in Gromacs.

## 3. Results and Discussion

### 3.1. Disordered Protein Prediction

**Figure 1:**
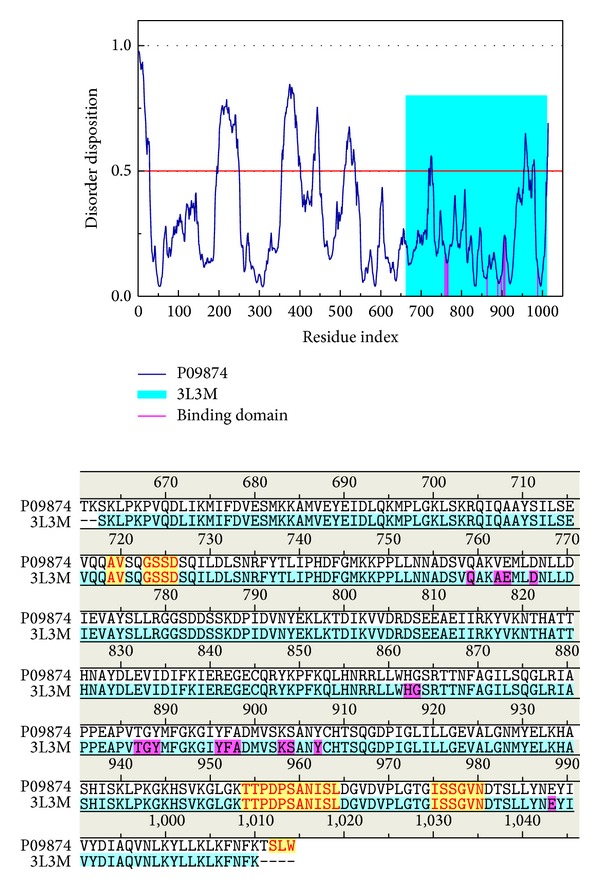
Disordered protein predicted by PONDR-Fit and sequence alignment with disordered residues (yellow regions) and residues in the binding domain (magenta regions).

The disordered amino acids of PARP-1 protein were predicted by PONDR-Fit with the protein sequence from Swiss-Prot (UniProtKB: P09874).  Figure 1 displays the result of disordered amino acids prediction and the sequence alignment. It indicates that the residues in the binding domain do not deposit in the disordered region. The binding domain of PARP-1 protein may have a stable structure in protein folding. Most residues in the binding domain were close to the local lowest regions of disordered disposition.

### 3.2. Docking Simulation

After virtual screening, the top TCM compounds ranked by dock score [46] and control, A927929, are listed in Table 1 with the results of three scoring functions, LigScore2 Dreiding [50], -PLP1 [51], -PLP2 [52], and -PMF [53].

LigScore2 Dreiding is a scoring function calculated by three descriptors as equation as follows:

(1) LigScore2_Dreiding=1.539−0.07622∗vdW +0.6501∗C+_pol−0.00007821 ×BuryPol2,

where *vdW* is a softened Lennard-Jones 6–9 potential in units of kcal/mol. C+_pol shows the buried polar surface area between protein and ligand in units of Å^2^. BuryPol^2^ is the squared sum of the buried polar surface area between protein and ligand in units of Å^2^.

-PLP1, -PLP2, and -PMF are calculated by summing pairwise interaction, which are hydrogen bond (H-bond) and steric interaction, between protein and ligand. Higher scores indicate stronger protein-ligand binding affinities.

**Figure 2:**
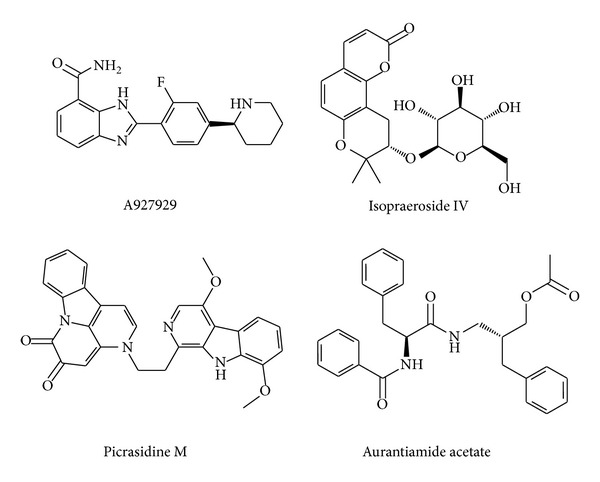
Chemical scaffolds of control and top three candidates.

**Figure 3:**
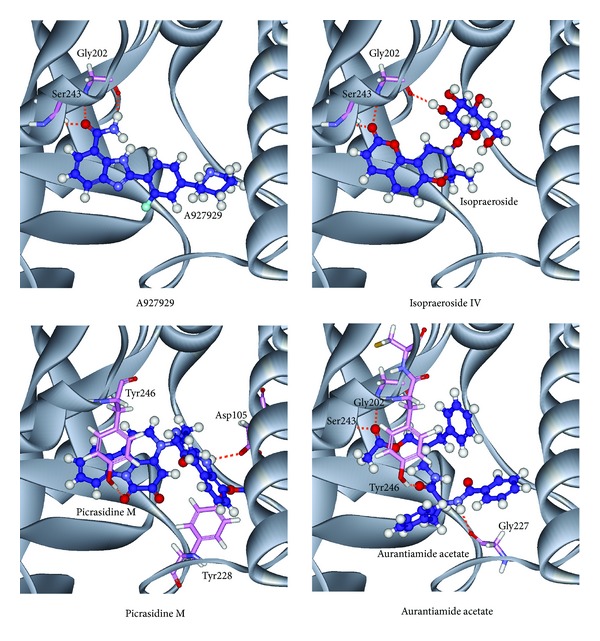
Docking poses of PARP-1 protein complexes with A927929, isopraeroside IV, picrasidine M, and aurantiamide acetate.

The scoring functions indicate that the top TCM compounds have higher binding affinities than A927929. The resources of three TCM compounds are also listed in Table 1. Isopraeroside IV is extracted from root of* Angelica dahurica*. Picrasidine M is extracted from bark of* Picrasma quassioides* (D.Don) Benn. Aurantiamide acetate is extracted from plant of* Artemisia annua* L. The chemical scaffolds of A927929 and top three TCM compounds are shown in Figure 2. The docking poses of A927929 and top TCM compounds in PARP-1 protein are illustrated in Figure 3. A927929 has H-bonds with two key residues Gly202 and Ser243, which restricted ligand in the binding domain. The TCM compounds, isopraeroside IV and aurantiamide acetate, have H-bonds with two key residues Gly202 and Ser243 as A927929. Moreover, aurantiamide acetate also has an H-bond with residue Gly227. Picrasidine M has H-bonds with another three residues Asp105, Tyr228, and Tyr246 to restricted ligand in the binding domain of PARP-1 protein.

### 3.3. Molecular Dynamics Simulation

**Figure 4:**
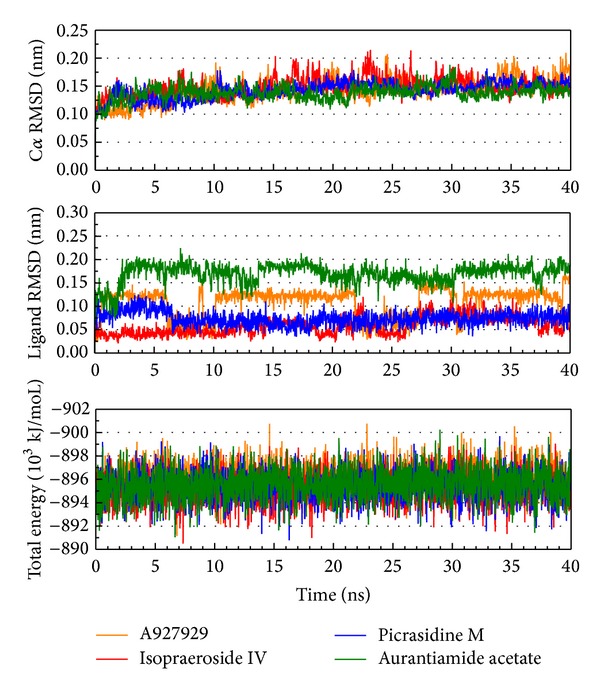
Root-mean-square deviation and total energy over 40 ns MD simulation for PARP-1 protein complexes with A927929, isopraeroside IV, picrasidine M, and aurantiamide acetate.

**Figure 5:**
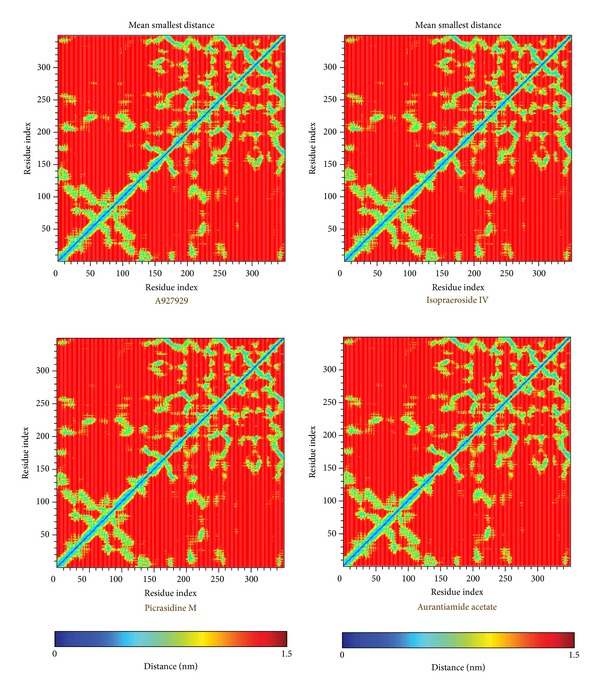
Distance matrices consisting of the smallest distance between residue pairs for PARP-1 protein complexes with A927929, isopraeroside IV, picrasidine M, and aurantiamide acetate. Residues 1–348 in *y*-axis correspond to residues 2–349.

**Figure 6:**
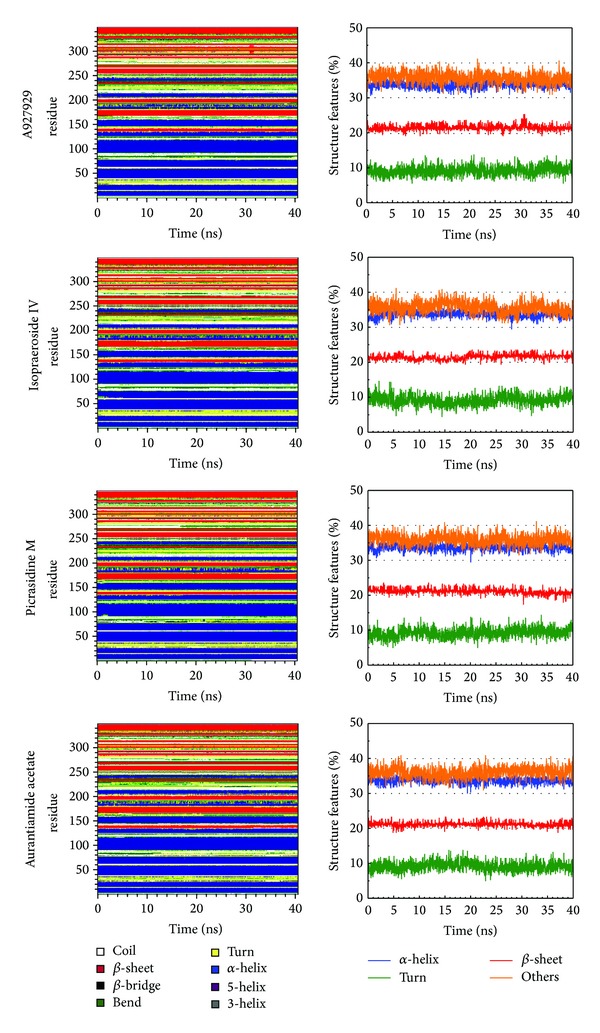
Secondary structure assignment and secondary structural feature ratio variations of each PARP-1 complex over 40 ns MD simulation. Residues 1–348 in *y*-axis correspond to residues 2–349.

**Figure 7:**
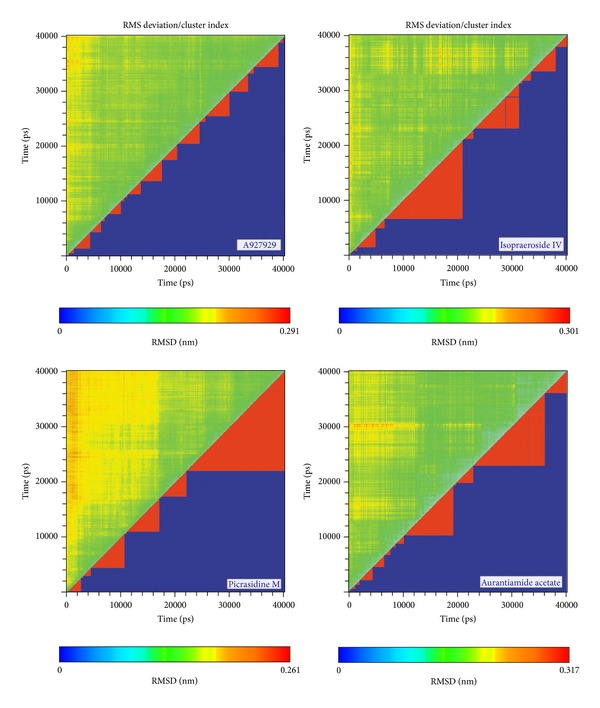
Root-mean-square deviation value (upper left half) and graphical depiction of the clusters with cutoff of 0.105 nm (lower right half) for PARP-1 protein complexes with A927929, isopraeroside IV, picrasidine M, and aurantiamide acetate.

**Figure 8:**
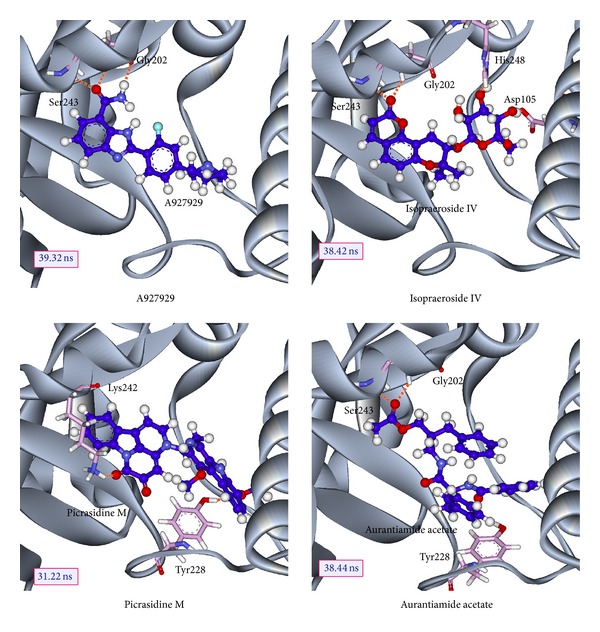
Docking poses of middle RMSD structure in the major cluster for PARP-1 protein complexes with A927929 (39.32 ns), isopraeroside IV (38.42 ns), picrasidine M (31.22 ns), and aurantiamide acetate (38.44 ns).

The molecular dynamics (MD) simulations were performed to analyze the stability of interactions between protein and ligand under dynamic conditions.  Figure 4 illustrates the root-mean-square deviations (RMSDs) and total energies for PARP-1 protein complexes with A927929, isopraeroside IV, picrasidine M, and aurantiamide acetate over 40 ns MD simulation. RMSDs were calculated to study atomic fluctuations for each protein and ligand during MD simulation. The C*α* RMSDs and ligand RMSDs indicate that each complex tends to stabilize after 31 ns of MD simulation. Moreover, Figure 4 also indicates that the PARP-1 complexes with the top three TCM compounds have similar total energies as the PARP-1 complex with A927929 under dynamic conditions. Distance matrices consisting of the smallest distance between residue pairs for each protein-ligand complex are shown in Figure 5. Those matrices display that the influence of the top three TCM compounds on the structure of PARP-1 protein is similar to A927929.  Figure 6 shows the secondary structure changes for each complex during MD simulation, respectively. The secondary structure changes indicate that the top three TCM compounds did not cause significant differences from the control. The secondary structural feature ratio variations indicate that each protein-ligand complex has approximately 33% of *α*-helix and 21% of *β*-sheet during MD simulation. In Figure 7, it illustrates the RMSD values and graphical depiction of the clusters with cutoff of 0.105 nm over 40 ns MD simulation. The RMSD values between MD trajectories indicate that the PARP-1 protein complexes tend to stabilize after MD simulation. After the complexes tend to stabilize under dynamic conditions, the representative structures of each protein-ligand complex after MD simulation were identified by middle RMSD structure in the major cluster. Docking poses of middle RMSD structure in the major cluster for PARP-1 protein complexes with A927929 (39.32 ns), isopraeroside IV (38.42 ns), picrasidine M (31.22 ns), and aurantiamide acetate (38.44 ns) are illustrated in Figure 8. It indicates that A927929 has a similar docking pose as docking simulation and maintains the H-bonds with two key residues Gly202 and Ser243 after MD simulation. For three TCM compounds, isopraeroside IV keeps the H-bonds with two key residues Gly202 and Ser243 under dynamic conditions. Moreover, isopraeroside IV has H-bonds with the other two residues Asp105 and His248 after MD simulation. Picrasidine M maintains the H-bond with residue Tyr228 under dynamic conditions and shifts an H-bond from residue Tyr246 to residue Lys242. In addition, picrasidine M loses the H-bond with residue Asp105 after MD simulation. Aurantiamide acetate maintains the H-bonds with two key residues Gly202 and Ser243 under dynamic conditions and has an H-bond with residue Tyr228 after MD simulation.

Docking poses of middle RMSD structure in the major cluster for PARP-1 protein complexes indicate that all compounds except picrasidine M have stable H-bonds with two key residues Gly202 and Ser243. Picrasidine M and aurantiamide acetate have an H-bond with residue Tyr228. Isopraeroside IV has H-bonds with the other two residues Asp105 and His248 after MD simulation.

**Figure 9:**
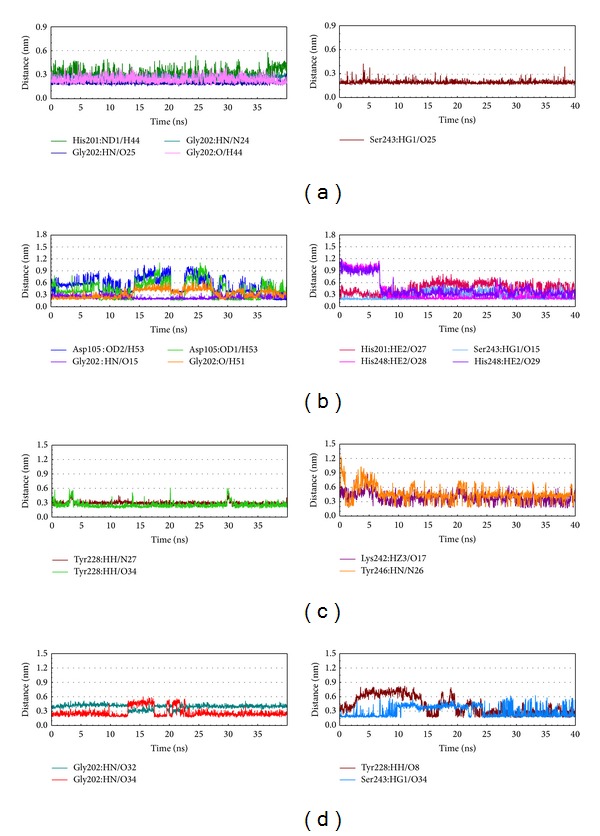
Distances of hydrogen bonds with common residues during 40 ns MD simulation. (a) A927929, (b) isopraeroside IV, (c) picrasidine M, and (d) aurantiamide acetate.

The occupancies of H-bonds for key residues of PARP-1 protein are listed in Table 2, and the fluctuation of distances for H-bonds with common residues of PARP-1 protein is shown in Figure 9. The H-bonds occupancies and distances fluctuation over MD simulation displays the stable H-bonds between ligands, A927929, isopraeroside IV, aurantiamide acetate, and residues Gly202 and Ser243. In addition, picrasidine M has stable H-bonds with residue Tyr228. For A927929, although the H-bond occupancy with residue His201 over 40 ns of MD simulation is 58%, the distance variation of H-bond shown in Figure 9 indicates that this H-bond was lost at the end of the MD simulation. For isopraeroside IV, the H-bonds with residues Asp105 and His248 are tended to stabilize after MD simulation. Aurantiamide acetate also has a stable H-bond with residue Tyr228 after 25 ns of MD simulation. For picrasidine M, the H-bond with residue Tyr246 in the docking simulation has shifted to binding with residue Lys242 after MD simulation, and it has another H-bond with residue Tyr246 under dynamic conditions.

The top TCM compounds, isopraeroside IV and aurantiamide acetate, have stable H-bonds with residues Gly202 and Ser243 as A927929. In addition, isopraeroside IV also has stable H-bonds with residues Asp105 and His248, which stabilized the docking pose of ligand in the binding domain. Aurantiamide acetate has another stable H-bond with residue Tyr228 similar to picrasidine M. For picrasidine M, it forms the stable H-bond with residue Lys242 instead of residues Gly202 and Ser243.

## 4. Conclusion

In this study, we aim to investigate the potent TCM candidates for PARP-1 protein. The top TCM candidates, isopraeroside IV, picrasidine M, and aurantiamide acetate had higher potent binding affinities than control, A927929, in the docking simulation. Both isopraeroside IV and aurantiamide acetate had H-bond with residues Gly202 and Ser243 as A927929. The MD simulations were performed to optimize the result of docking simulation and validate the stability of H-bonds between each ligand and PARP-1 protein under dynamic conditions. Isopraeroside IV and aurantiamide acetate have stable H-bonds with residues Gly202 and Ser243 as A927929 under dynamic conditions. Moreover, they had stable H-bonds with residues Asp105, Tyr228, and His248 in the other side of the binding domain, which may strengthen and stabilize ligand inside the binding domain of PARP-1 protein. Hence, we propose isopraeroside IV and aurantiamide acetate as potential lead compounds for further study in drug development process with the PARP-1 protein.

## Figures and Tables

**Table 1:** Scoring functions of top candidates and A927929 from TCM database screening.

Name	Resource	LigScore2 Dreiding	-PLP1	-PLP2	-PMF	Dock score
Isopraeroside IV	*Angelica dahurica *	6.42	122.67	116.48	163.17	100.596
Picrasidine M	*Picrasma quassioides *(D. Don) Benn.	6.92	125.68	121.49	162.36	92.256
Aurantiamide acetate	*Artemisia annua *L.	6.74	136.86	132.63	159.08	88.910
A927929*		**6.10**	**118.83**	**115.73**	**120.22**	** 52.093**

*Control.

**Table 2:** H-bond occupancy for key residues of PARP-1 protein with top three candidates and A927929 overall 40 ns molecular dynamics simulation.

Name	H-bond interaction		Occupancy
A927929	His201:ND1	/H44	58%
	Gly202:HN	/N24	88%
	Gly202:HN	/O25	100%
	Gly202:O	/H44	86%
	Ser243:HG1	/O25	100%

Isopraeroside IV	Asp105:OD1	/H53	32%
	Asp105:OD2	/H53	5%
	His201:HE2	/O27	17%
	Gly202:HN	/O15	87%
	Gly202:O	/H51	44%
	Ser243:HG1	/O15	63%
	His248:HE2	/O28	71%
	His248:HE2	/O29	22%

Picrasidine M	Tyr228:HH	/N27	66%
	Tyr228:HH	/O34	87%
	Lys242:HZ3	/O17	20%
	Tyr246:HH	/N26	11%

Aurantiamide acetate	Gly202:HN	/O32	6%
	Gly202:HN	/O34	78%
	Tyr228:HH	/O8	35%
	Ser243:HG1	/O34	55%

H-bond occupancy cutoff: 0.3 nm.

## References

[1] D’Amours D, Desnoyers S, D’Silva I, Poirier GG (1999). Poly(ADP-ribosyl)ation reactions in the regulation of nuclear functions. *The Biochemical Journal*.

[2] Bai P, Canto C (2012). The role of PARP-1 and PARP-2 enzymes in metabolic regulation and disease. *Cell Metabolism*.

[3] Hassa PO, Hottiger MO (2008). The diverse biological roles of mammalian PARPs, a small but powerful family of poly-ADP-ribose polymerases. *Frontiers in Bioscience*.

[4] Krishnakumar R, Kraus WL (2010). The PARP side of the nucleus: molecular actions, physiological outcomes, and clinical targets. *Molecular Cell*.

[5] Langelier MF, Pascal JM (2013). PARP-1 mechanism for coupling DNA damage detection to poly(ADP-ribose) synthesis. *Current Opinion in Structural Biology*.

[6] McCabe N, Turner NC, Lord CJ (2006). Deficiency in the repair of DNA damage by homologous recombination and sensitivity to poly(ADP-ribose) polymerase inhibition. *Cancer Research*.

[7] Langelier M-F, Servent KM, Rogers EE, Pascal JM (2008). A third zinc-binding domain of human poly(ADP-ribose) polymerase-1 coordinates DNA-dependent enzyme activation (Journal of Biological Chemistry (2008) 283, (4105–4114)). *The Journal of Biological Chemistry*.

[8] Altmeyer M, Messner S, Hassa PO, Fey M, Hottiger MO (2009). Molecular mechanism of poly(ADP-ribosyl)ation by PARP1 and identification of lysine residues as ADP-ribose acceptor sites. *Nucleic Acids Research*.

[9] Tao ZH, Gao P, Liu H-W (2009). Identification of the ADP-ribosylation sites in the PARP-1 automodification domain: analysis and implications. *Journal of the American Chemical Society*.

[10] Langelier MF, Planck JL, Roy S, Pascal JM (2012). Structural basis for DNA damage-dependent poly(ADP-ribosyl)ation by human PARP-1. *Science*.

[11] Ruf A, de Murcia G, Schulz GE (1998). Inhibitor and NAD+ binding to poly(ADP-ribose) polymerase as derived from crystal structures and homology modeling. *Biochemistry*.

[12] Ruf A, de Murcia JM, de Murcia GM, Schulz GE (1996). Structure of the catalytic fragment of poly(ADP-ribose) polymerase from chicken. *Proceedings of the National Academy of Sciences of the United States of America*.

[13] Kameshita I, Matsuda Z, Taniguchi T, Shizuta Y (1984). Poly(ADP-ribose) synthetase. Separation and identification of three proteolytic fragments as the substrate-binding domain, the DNA-binding domain, and the automodification domain. *The Journal of Biological Chemistry*.

[14] Spigel DR (2012). PARP inhibitors in lung cancer. *Journal of Thoracic Oncology*.

[15] Reinbolt RE, Hays JL (2013). The role of PARP inhibitors in the treatment of gynecologic malignancies. *Frontiers in Oncology*.

[16] Farmer H, McCabe H, Lord CJ (2005). Targeting the DNA repair defect in BRCA mutant cells as a therapeutic strategy. *Nature*.

[17] Fong PC, Boss DS, Yap TA (2009). Inhibition of poly(ADP-ribose) polymerase in tumors from BRCA mutation carriers. *The New England Journal of Medicine*.

[18] Lin D-Y, Tsai F-J, Tsai C-H, Huang C-Y (2011). Mechanisms governing the protective effect of 17*β*-estradiol and estrogen receptors against cardiomyocyte injury. *Biomedicine*.

[19] Liao W-L, Tsai F-J (2013). Personalized medicine: a paradigm shift in healthcare. *BioMedicine*.

[20] Lee C-C, Tsai C-H, Wan L (2013). Increased incidence of Parkinsonism among Chinese with *β*-glucosidase mutation in central Taiwan. *BioMedicine*.

[21] Wang C-H, Lin W-D, Bau D-T (2013). Appearance of acanthosis nigricans may precede obesity: an involvement of the insulin/IGF receptor signaling pathway. *BioMedicine*.

[22] Chang Y-M, Velmurugan BK, Kuo WW (2013). Inhibitory effect of alpinate Oxyphyllae fructus extracts on Ang II-induced cardiac pathological remodeling-related pathways in H9c2 cardiomyoblast cells. *BioMedicine*.

[23] Chou IC, Lin W-D, Wang C-H (2013). Association analysis between Tourette's syndrome and two dopamine genes (DAT1, DBH) in Taiwanese children. *BioMedicine*.

[24] Lin WY, Liu HP, Chang JS (2013). Genetic variations within the PSORS1 region affect Kawasaki disease development and coronary artery aneurysm formation. *BioMedicine*.

[25] Chen C-Y, Chang Y-H, Bau D-T (2009). Ligand-based dual target drug design for H1N1: swine flu—a preliminary first study. *Journal of Biomolecular Structure and Dynamics*.

[26] Chang S-S, Huang H-J, Chen CY-C (2011). Two birds with one stone? Possible dual-targeting H1N1 inhibitors from traditional Chinese medicine. *PLoS Computational Biology*.

[27] Chang T-T, Sun M-F, Chen H-Y (2011). Screening from the world’s largest TCM database against H1N1 virus. *Journal of Biomolecular Structure and Dynamics*.

[28] Lin C-H, Chang T-T, Sun M-F (2011). Potent inhibitor design against H1N1 swine influenza: structure-based and molecular dynamics analysis for M2 inhibitors from traditional Chinese medicine database. *Journal of Biomolecular Structure and Dynamics*.

[29] Chen K-C, Yu-Chian Chen C (2011). Stroke prevention by traditional Chinese medicine? A genetic algorithm, support vector machine and molecular dynamics approach. *Soft Matter*.

[30] Chen K-C, Chang K-W, Chen H-Y, Chen CY-C (2011). Traditional Chinese medicine, a solution for reducing dual stroke risk factors at once?. *Molecular BioSystems*.

[31] Chang T-T, Chen K-C, Chang K-W (2011). In silico pharmacology suggests ginger extracts may reduce stroke risks. *Molecular BioSystems*.

[32] Tsou YA, Chen KC, Lin HC, Chang SS, Chen CYC (2012). Uroporphyrinogen decarboxylase as a potential target for specific components of traditional Chinese medicine: a virtual screening and molecular dynamics study. *PLoS ONE*.

[33] Yang S-C, Chang S-S, Chen H-Y, Chen CY-C (2011). Identification of potent EGFR inhibitors from TCM Database@Taiwan. *PLoS Computational Biology*.

[34] Chen C-Y, Chen CY-C (2010). Insights into designing the dual-targeted HER2/HSP90 inhibitors. *Journal of Molecular Graphics and Modelling*.

[35] Huang H-J, Lee K-J, Yu HW (2010). Structure-based and ligand-based drug design for HER 2 receptor. *Journal of Biomolecular Structure and Dynamics*.

[36] Huang H-J, Lee K-J, Yu HW, Chen H-Y, Tsai F-J, Chen CY-C (2010). A novel strategy for designing the selective PPAR agonist by the “Sum of activity” model. *Journal of Biomolecular Structure and Dynamics*.

[37] Chen KC, Chang SS, Huang HJ (2012). Three-in-one agonists for PPAR-alpha, PPAR-gamma, and PPAR-delta from traditional Chinese medicine. *Journal of Biomolecular Structure & Dynamics*.

[38] Chang P-C, Wang J-D, Lee M-M (2011). Lose weight with traditional Chinese medicine? Potential suppression of fat mass and obesity-associated protein. *Journal of Biomolecular Structure and Dynamics*.

[39] Tou WI, Chen CY (2013). May disordered protein cause serious drug side effect?. *Drug Discovery Today*.

[40] Chen CY, Tou WI (2013). How to design a drug for the disordered proteins?. *Drug Discovery Today*.

[41] Penning TD, Zhu G-D, Gong J (2010). Optimization of phenyl-substituted benzimidazole carboxamide poly(ADP-ribose) polymerase inhibitors: identification of (S)-2-(2-fluoro-4- (pyrrolidin-2-yl)phenyl)-1 H -benzimidazole-4-carboxamide (A-966492), a highly potent and efficacious inhibitor. *Journal of Medicinal Chemistry*.

[42] Brooks BR, Bruccoleri RE, Olafson BD (1983). CHARMM: a program for macromolecular energy minimization and dynamics calculations. *Journal of Computational Chemistry*.

[43] Chen CY-C (2011). TCM database@Taiwan: the world’s largest traditional Chinese medicine database for drug screening in Silico. *PLoS ONE*.

[44] Lipinski CA, Lombardo F, Dominy BW, Feeney PJ (2001). Experimental and computational approaches to estimate solubility and permeability in drug discovery and development settings. *Advanced Drug Delivery Reviews*.

[45] Xue B, Dunbrack RL, Williams RW, Dunker AK, Uversky VN (2010). PONDR-FIT: a meta-predictor of intrinsically disordered amino acids. *Biochimica et Biophysica Acta—Proteins and Proteomics*.

[46] Venkatachalam CM, Jiang X, Oldfield T, Waldman M (2003). LigandFit: a novel method for the shape-directed rapid docking of ligands to protein active sites. *Journal of Molecular Graphics and Modelling*.

[47] Hess B, Kutzner C, van der Spoel D, Lindahl E (2008). GRGMACS 4: algorithms for highly efficient, load-balanced, and scalable molecular simulation. *Journal of Chemical Theory and Computation*.

[48] Zoete V, Cuendet MA, Grosdidier A, Michielin O (2011). SwissParam: a fast force field generation tool for small organic molecules. *Journal of Computational Chemistry*.

[49] Fletcher R (1969). *Optimization*.

[50] Mayo SL, Olafson BD, Goddard WA (1990). DREIDING: a generic force field for molecular simulations. *Journal of Physical Chemistry*.

[51] Gehlhaar DK, Verkhivker GM, Rejto PA (1995). Molecular recognition of the inhibitor AG-1343 by HIV-1 protease: conformationally flexible docking by evolutionary programming. *Chemistry and Biology*.

[52] Gehlhaar Daniel K, Bouzida D, Rejto Paul A (1999). Reduced dimensionality in ligand? Protein structure prediction: covalent inhibitors of serine proteases and design of site-directed combinatorial libraries. *Rational Drug Design*.

[53] Muegge I, Martin YC (1999). A general and fast scoring function for protein-ligand interactions: a simplified potential approach. *Journal of Medicinal Chemistry*.

